# Prophages and adaptation of *Staphylococcus aureus* ST398 to the human clinic

**DOI:** 10.1186/s12864-017-3516-x

**Published:** 2017-02-06

**Authors:** Seydina M. Diene, Anna Rita Corvaglia, Patrice François, Nathalie van der Mee-Marquet, on behalf of the Regional Infection Control Group of the Centre Region

**Affiliations:** 1 Genomic Research Laboratory, Service of Infectious Diseases, Geneva University Hospitals, Geneva, Switzerland; 2 Département de Microbiologie, Centre Hospitalier Régional Universitaire, and UMR 1282 Infectiologie Santé Publique, Université François-Rabelais, Tours, France

**Keywords:** *Staphylococcus aureus*, CC398 lineage, Phage content, Prophage, Evolution, Livestock-associated, φ3-prophage, Bloodstream infections

## Abstract

**Background:**

It has been suggested that prophages in the ST398 *S. aureus* clone are responsible for expanding ST398's spectrum of action and increasing its ability to cause human infections. We carried out the first characterization of the various prophages carried by 76 ST398 bloodstream infection (BSI) isolates obtained over 9 years of observation.

**Results:**

Whole-genome sequencing of 22 representative isolates showed (1) the presence of the φ3-prophage and diverse genetic features typical of animal-associated isolates (i.e., *SCCmec* XI element, Tn916 transposon and non φ3-prophages) in a majority of BSI isolates, (2) one BSI isolate devoid of the φ3-prophage but otherwise similar to an animal-infecting isolate, (3) 35 prophages carrying numerous genes previously associated with virulence or immune evasion in animal models of staphylococcal infections. The analysis of prophage content in all 76 BSI isolates showed an increasing prevalence of polylysogeny over time. Overall, over the course of the last 10 years, the BSI isolates appear to have acquired increasing numbers of genetic features previously shown to contribute to bacterial adaptation and virulence in animal models of staphylococcal infections.

**Conclusions:**

We hypothesize that lysogeny has played a significant role in increasing the ability of the ST398 clone to cause infections in humans. Our findings highlight the risk that the ST398 lineage will increase its threat to public health by continuing to acquire virulence and/or multiple antibiotic-resistance genes from hospital-associated clones of *Staphylococcus aureus*.

**Electronic supplementary material:**

The online version of this article (doi:10.1186/s12864-017-3516-x) contains supplementary material, which is available to authorized users.

## Background

*Staphylococcus aureus* sequence type 398 (ST398) is a lineage initially described in the early 2000s in colonized livestock pigs and in humans living in close contact with these animals [1, 2]. Evidence for two major evolutionary changes in the ST398 lineage was recently provided by studies conducted worldwide [3–9]. First, there has been a widening of the infection spectrum of these bacteria to include humans living in animal-free environments and companion and livestock animals other than pigs. Second, ST398’s intrinsic virulence capacity has increased enabling it to cause a larger number of invasive infections in humans, such as bloodstream infections (BSIs), endocarditis and osteo-articular infections.

Through an active BSI surveillance program conducted since 2000 in a cohort of hospitals [10], we identified, in 2007, the first cases of ST398-BSIs in French patients living in animal-free environments [7]. ST398 has since become established as a major *S. aureus* clone responsible for BSIs in the analyzed area. The incidence of ST398-BSIs increased from 0.079 cases/100,000 inhabitants/3 months in 2007 to 0.853 cases/100,000 inhabitants/3 months in 2015. The ST398 clone accounted for 15% of MSSA isolates responsible for BSI in 2015, whereas it had never been detected in BSI cases before 2007.

The molecular mechanisms driving the adaptation of the ST398 lineage in humans remain unclear. Multi-locus sequence typing (MLST) indicated that strains are “clonal”, and differ only by their respective content in mobile genetic elements. Therefore, this “clonal” population differs drastically in terms of phenotypic features. Some studies have suggested a key role for prophages in the epidemiological changes currently taking place in this lineage [8, 11, 12]. However, the prophages carried by the human-adapted isolates remain poorly characterized. Here, we analyzed the prophage content of a complete cohort of ST398-BSI isolates recovered during a continuous study carried out over a period of 15 years in the same health centers [10]. We identified and characterized the prophage features carried by BSI isolates, and tracked how the prophages changed over time. We also used a whole-genome sequencing approach to analyze the prophages carried by representative BSI isolates recovered during the study period, and to compare these prophage genomes with those carried by ST398 isolates that colonize and infect animals. The evidence of on-going prophage acquisition by the BSI isolates between 2007 and 2015 and the consequent increase of embedded genetic features likely to mediate adaptation to humans, provide new insight into the rapid evolution of the ST398 lineage.

## Methods

### Bacterial isolates

We studied 76 *S. aureus* isolates recovered from patients diagnosed with ST398-BSI in the course of a previously described survey [10]. The collection contains one MRSA and 75 MSSA (98.7%) isolates, most of which were susceptible to all the antibiotics tested, with the exception of erythromycin (84.2% of the isolates were resistant to this antibiotic). Most isolates were *spa*-type t571 or t1451 (50.7 and 30.4%, respectively); the MRSA isolate was *spa*-type t899. For comparative analysis, we used five ST398 animal-associated isolates representative of the isolates colonizing and infecting animals described in previous studies [6, 7, 12–15], and the animal-associated MRSA isolate s0385, which was responsible for a human case of endocarditis [16].

### Genome sequencing

High-throughput sequencing technology was used to sequence the genomes of 22 ST398 isolates, including 16 BSI isolates randomly selected from our collection, and the six isolates representative of animal-associated isolates described in previous studies [6, 12]. Genomic DNA from each isolate was purified on DNeasy columns (Qiagen), and then sequenced on an Illumina HiSeq 2500 (Illumina, San Diego, CA, USA), with 100-base paired-end reads and barcodes within the Nextera XT kit (Illumina), used in accordance with the manufacturer’s instructions. Read sequence quality was assessed with the Fastqc program (http://www.bioinformatics.babraham.ac.uk/projects/fastqc/) and reads were quality-filtered with fastq-mcf (Ea-utils: https://expressionanalysis.github.io/ea-utils/). Genome assembly was performed with the Edena v3 assembler [17]. The assembled genomes were annotated with Prokka v1.10 software [18]. The phylogenetic relationships between isolates were investigated by genomic single-nucleotide polymorphism (SNP) analysis with Parsnp v1.0 software [19]. We used “CGView Comparison Tool” software [20] to compare the proteomes of the isolates. Prophage sequences were identified from the genome sequences by blast with the PHAST database [21] and the “Get_homologues.pl” script was used for core proteome comparisons [22]. The CVTree3 Web Server was used to perform the phylogenetic analysis between the prophages identified in our isolates [23]. This program uses whole-genome data without sequence alignment to infer the phylogenetic tree through a Composition Vector (CV) approach [23]. Prokka annotation and blastP analysis were performed to identify specific genes involved in the phenotypes, evolution, and virulence of the isolates. Predicted gene functions were based on protein similarities at least of 40% and E-values less than or equal to 10^−6^.

### PCR detection of prophage elements in each isolate

We characterized the prophage content of the human and animal isolates using a multiplex PCR-based multilocus diagnostic scheme described by Kahankova that is based on a broad diversity of staphylococcal phages [23]. This scheme is a molecular tool for detecting, within bacterial genomes, DNA fragments that are similar to prophage genes encoding integrases, antirepressors, replication proteins, dUTPases, portal proteins, tail appendices and endolysins [24].

## Results

### Phylogenetic analysis of core-genome SNPs distinguishes between BSI isolates and animal-associated isolates

**Fig. 1:**
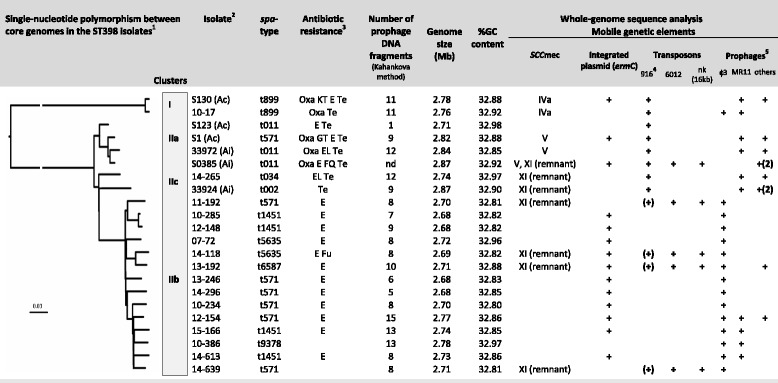
General results of the whole-genome sequence analysis. ^1^ Single-nucleotide polymorphism between core genomes in the human and animal ST398 isolates. In this comparison, 14–265 is used as the reference genome; ^2^(Ac) colonizing animal-associated isolate, (Ai) infecting animal-associated isolate; all other isolates are from human BSI ^3^Oxa (methicillin), K (kanamycin), T (tobramycin), E (erythromycin), Te (tetracycline), Fu (fusidic acid); ^4^(+) Tn916-like element, with no *tet* gene; ^5^ + (2) two different prophages harboured into the bacterial genome

The core genome SNPs identified in the genomes of 22 ST398 isolates representative of human and animal isolates were used to infer a phylogenetic tree, which indicated two divisions of isolates (Fig. 1): Division I contained the two t899 isolates, and Division II comprised 3 clusters: IIa containing only animal-associated isolates, IIb containing only BSI isolates and IIc containing one BSI isolate and one animal infecting isolate. The very similar core genomes of all BSI isolates except one (isolate 14–265 in cluster IIc) suggests recent evolution of the human-adapted ST398 subpopulation.

### Animal-associated accessory genetic features in the genome of BSI isolates

**Fig. 2:**
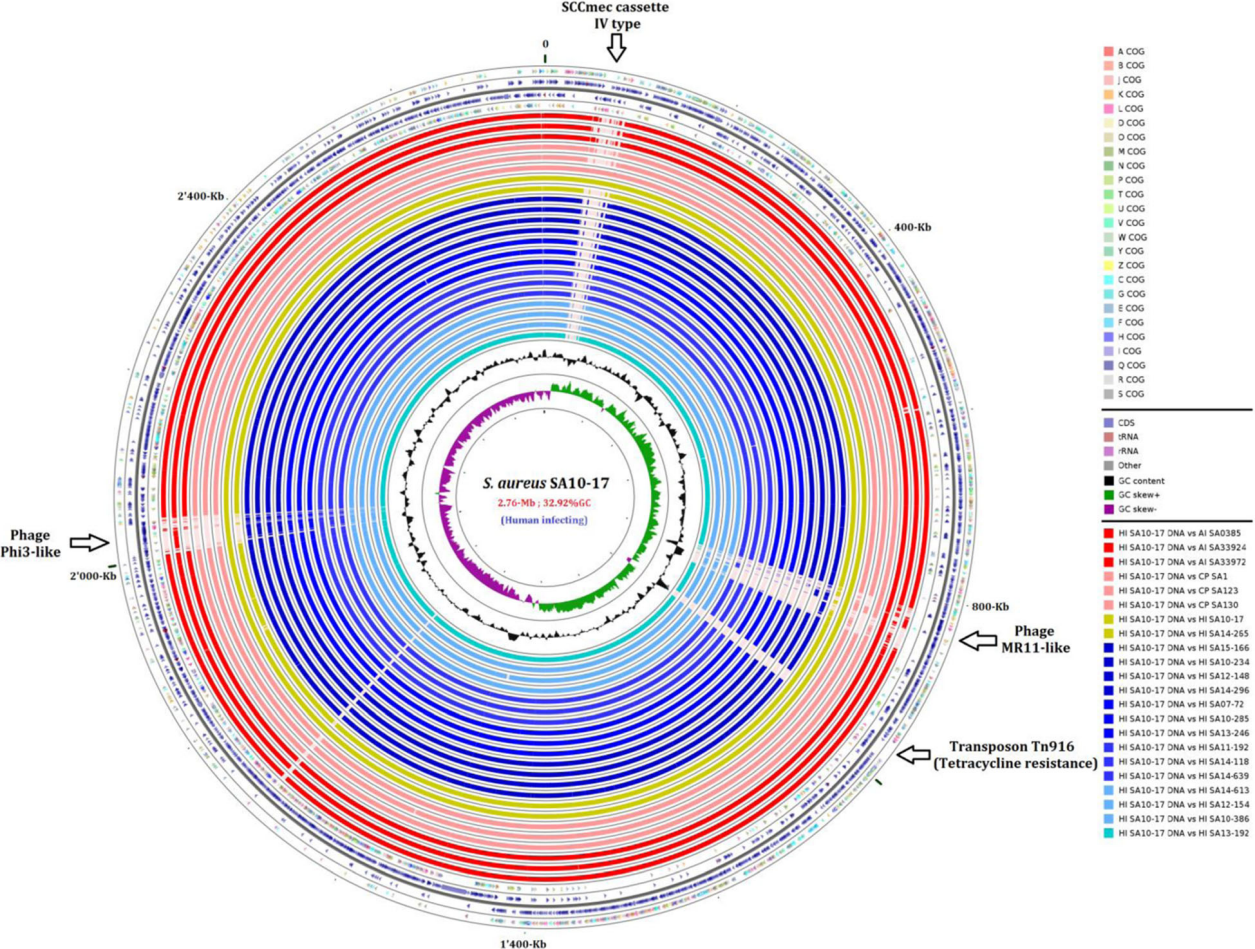
Comparisons of the proteomes of animal-associated infecting (AI, in red), animal-associated colonizing (AC, in light red), and human BSI-associated (from dark to light blue) *S. aureus* ST398 isolates. This figure was created with the “CGView Comparison Tool” [21]. From the outside to the inside: ring 1, COG classification (see COG definitions in Additional file 4: Table S1) of ORFs shown in ring 2; rings 2 and 3, ORFs; ring 4, COG classification of ORFs shown in ring 3; rings 5 to 10 (in red and light red), the AI and AC genomes; rings 11 and 12 (in dark yellow), the reference genome (HI SA10-17 isolate) and the unusual HI SA14_265 genome; rings 13 to 26 (in blue), all other HI genomes; rings 27 and 28, the %GC content and GC skew, respectively, of the reference genome. Arrows indicate the locations of the φ3-like and MR11-like prophages, the transposon Tn916, and the SCCmec IV type in the SA10-17 genome

The pool of accessory genes differed considerably between BSI isolates, excluding the possibility of a single epidemic due to the spread of clonal isolates. Multiple mobile genetic elements (MGEs), including 35 prophages were identified in the genomes of the isolates studied (Table 1, Figs. 1 and 2, Additional file 1: Table S2). φ3-prophages were the only MGEs carried by most (15/16) BSI isolates and these prophages were absent from animal-associated isolates. By contrast, the other MGEs (i.e., non φ3-prophages, *SSCmec* elements and transposons) were carried by all, or almost all the animal-associated isolates (Figs. 1 and 2). However, these animal-associated MGEs were also found in some BSI isolates: (1) the *SSCmec* IVa type was found in isolate 10–17, (2) remnants of *SCCmec* XI, a genetic element mostly found in *S. aureus* isolates responsible for bovine mastitis [25], were identified in six BSI isolates, (3) a *Tn*916-like transposon encoding *tet* gene was found in two isolates, and remnants of a *Tn*916-like transposon were found in four other isolates, and (4) non φ3-prophages were detected in 43.7% of the BSI isolates. Most BSI isolates had at least one genetic feature typical of animal-associated MGEs in their genome (71.4%), suggesting that these isolates may have originated in an animal setting.

**Table 1:** Major characteristics of the 35 prophages carried by the 22 ST398 isolates studied

Phage names	Cluster	Ac. N° of the host genome	Size (bp)	%G + C	Number of CDSs
10–285 phi01	φ3	LNJH00000000	47′596	32.76	62
15–166 phi02	φ3	LXGQ00000000	47′598	32.76	64
14–296 phi01	φ3	LXGS00000000	47′598	32.76	64
12–148 phi01	φ3	LXGP00000000	47′595	32.76	64
10–234 phi01	φ3	LXGR00000000	76′003	31.38	93
10–386 4pro	φ3	AUPV0100000	42′906	33.08	59
14–613 phi02	φ3	LNJN00000000	47′149	32.76	62
14–639 phi01	φ3	LNJO00000000	47′598	32.75	62
13–246 phi01	φ3	LNJK00000000	47′598	32.76	62
07–72 phi01	φ3	AUPW0100000	51′402	32.68	67
13–192 phi02	φ3	LNJF00000000	46′241	32.67	61
12–154 phi02	φ3	LNJJ00000000	31′531	31.90	41
14–118 phi01	φ3	LNJL00000000	47′581	32.75	62
11–192 phi01	φ3	LNJI00000000	48′616	32.71	65
10–17 phi02	φ3	LNJG00000000	71′567	32.01	69
14–613 phi01	MR11-like	LNJN00000000	60′024	34.61	77
15–166 phi01	MR11-like	LXGQ00000000	59′797	34.63	80
10–386 5pro	MR11-like	AUPV0100000	43′301	35.14	66
12–154 phi01	MR11-like	LNJJ00000000	46′683	34.95	59
33972 phi01	MR11-like	LNKX00000000	60′070	35.55	74
10–17 phi01	MR11-like	LNJG00000000	69′195	34.16	83
14–265 phi02	MR11-like	LNJM00000000	50′513	35.04	66
s130 phi02	MR11-like	AUPT0100000	48′363	34.69	75
s1 3pro	MR11-like	AUPS0100000	41′392	35.60	67
33924 phi01	MR11-like	LNKW00000000	42′831	35.53	69
s0385 phi01	Others	NC_017333	48′968	33.73	68
s03985 phi02	Others	NC_017333	72′587	33.13	75
s1 2pro	Others	AUPS0100000	45′572	33.34	61
s130 phi01	Others	AUPT0100000	48′456	33.69	64
33972 phi02	Others	LNKX00000000	72′917	33.1	72
33924 phi02	Others	LNKW00000000	61′369	33.66	100
33924 phi03	Others	LNKW00000000	36′575	33.48	59
14–265 phi01	Others	LNJM00000000	48′081	33.96	65
12–154 phi03	Others	LNJJ00000000	56′681	34.15	76
13–192 phi01	Others	LNJF00000000	42′303	34.30	62

### Prophage-encoded loci known to contribute to bacterial pathogenesis

**Fig. 3:**
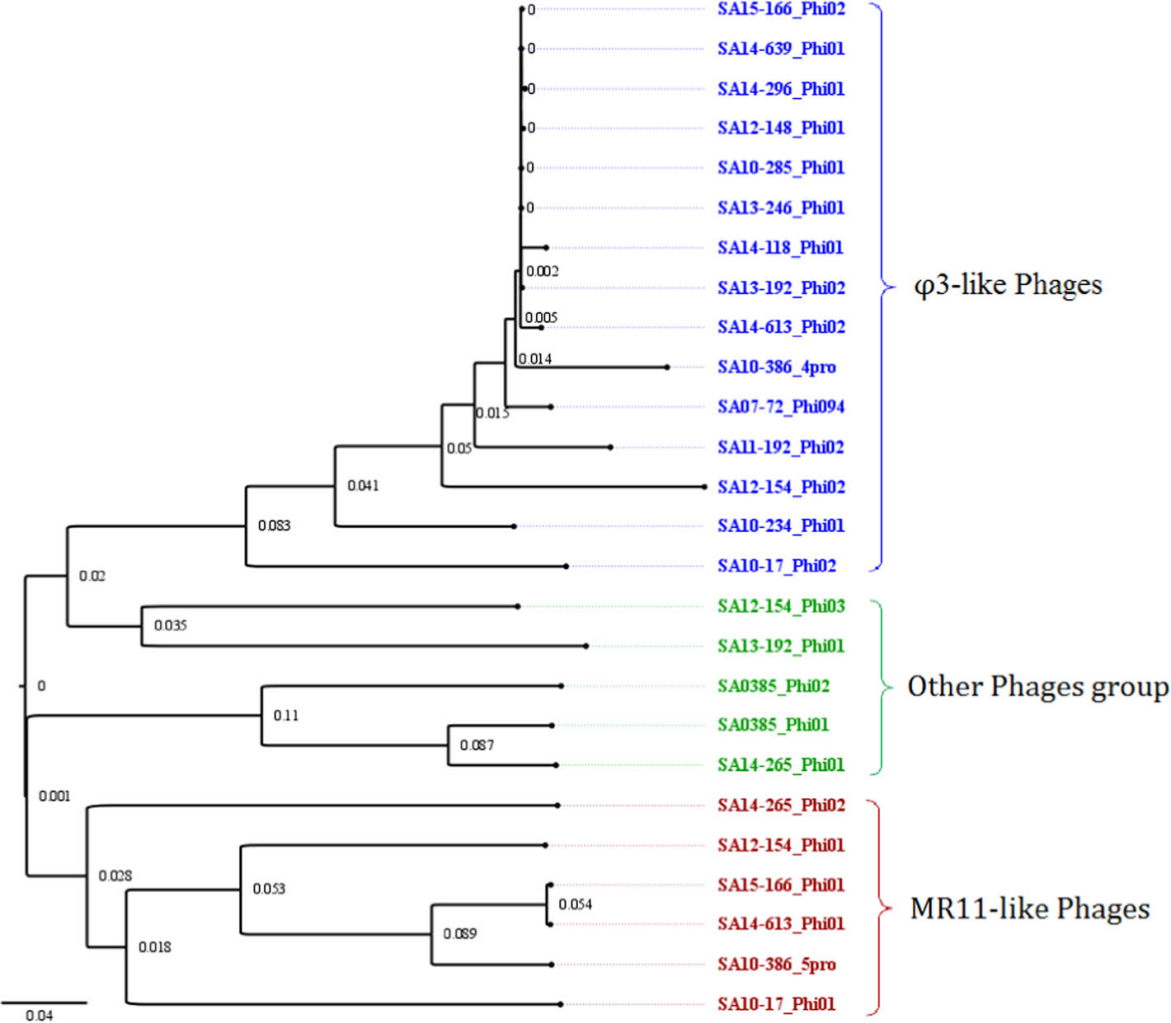
Proteome-based clustering of the prophages harbored by BSI isolates. The tree was inferred using the online web-server “CVTree3” program (http://tlife.fudan.edu.cn/cvtree3/). Branch lengths referring to the sequence variations are shown on the tree

The sequences of the 35 prophages identified in the genomes of the 22 ST398 isolates were analyzed (Table 1). A comparison of these sequences defined three groups (Fig. 3; Additional file 2: Figure S1 and Additional file 3: Figure S2). The first group comprised homogeneous φ3-prophages (Additional file 3: Figure S2) integrated in the genome by interrupting the phospholipase C gene that encodes a β-hemolysin. Each of these phages carried a set of genes encoding putative virulence factors: the small secreted proteins (Chp and Scn) involved in immune evasion, a leukocidin-like protein, the small RNA (named SprD) that has been shown to significantly contribute to disease in an animal model [26], a tyrosine recombinase (XerC) linked to biofilm-associated staphylococcal infections and acute bacteremia [27], and an ATP-dependent Clp protease that has been shown to have a major impact on virulence, the stress response and physiology in *S. aureus* [28] (Table 2). The φ3-prophages also harbored a gene encoding an HNH endonuclease involved in a putative defense system, and a LexA/antirepressor protein KilAC-mediated mechanism involved in synchronization of prophage induction in polylysogenic strains and prompt reestablishment of lysogeny and viral replication following prophage induction [29]. See Additional file 4 for COG categories.

**Table 2:**
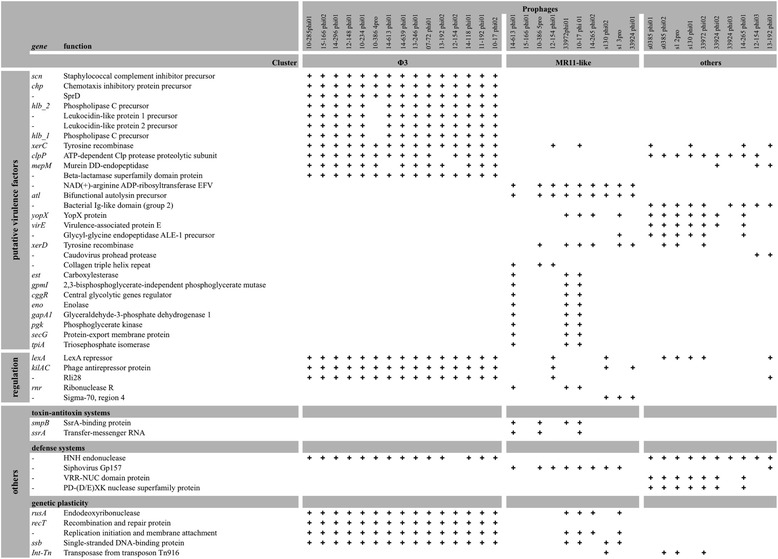
Characterization of the genomes of 35 prophages encoding putative factors related to virulence, the regulation of transcription, toxin-antitoxin systems, defense systems and genetic plasticity

The second group of prophages had genomes similar to the MR11 phage. These prophages mostly carried genes encoding an ADP-ribosyltransferase toxin [30], a putative phage defense mechanism, and an autolysin Atl involved in regulating cell wall growth, stress-induced autolysis and bacterial pathogenesis through the release of cell-wall components or by mediating adhesion to host tissue [31]. Three MR11-like prophages also carried a putative SsrA-SmpB toxin-antitoxin system [32], and a complete glycolytic operon comprising the *gpml*, *cggR*, *eno*, *pgk*, *tpiA* and *gapA* genes. These genes have been shown to play an important role in *S. aureus* infection in an invertebrate model [33].

The third group contained various prophages that mostly contains genes encoding the virulence-associated protein VirE, the lysostaphin-like glycyl-glycine endopeptidase ALE-1 [34], a bacterial Ig-like domain [35], putative restriction modification systems, and a type III secretion system YopX protein shown to modulate host cell signaling responses and pathogenesis [36]. Like φ3-prophages, these prophages also had genes encoding an ATP-dependent Clp protease. These data indicate that lysogenic ST398 isolates have acquired a considerable number of loci known to contribute to bacterial pathogenesis, particularly in the case of isolates harboring φ3-prophages, but also in the case of polylysogeny.

### Increase in the prevalence of polylysogenic BSI isolates over the last 10 years

The prophage content of all 76 BSI isolates was studied by the Kahankova method that uses specific primers to target sequences of bacteriophage origin [24]. We detected a total of 793 prophage DNA fragments (Ps) (Additional file 1: Table S2), and the number of Ps per BSI isolate ranged from 4 to 17 (mean value: 10). The mean number of Ps per isolate increased from 8 in 2007 to 12 in 2015. Isolates with a maximum of 10 Ps were significantly associated with BSIs diagnosed before 2012, whereas isolates containing more than 10 Ps were significantly more prevalent after 2012 (*p* = 0.006). Whole-genome sequencing (WGS) analysis of the 22 ST398 isolates showed that having no more than 10 prophage DNA fragments detectable by the Kahankova method was significantly associated with the carriage of a single prophage, whereas the isolates with more than 10 Ps were polylysogenic (*p* < 0.001). Thus, given the increase in the mean number of Ps per isolate during the study period, our findings indicate that the prevalence of polylysogenic BSI isolates increased significantly between 2007 and 2015.

## Discussion

It has been suggested that prophages played a role in the rapid evolution of the ST398 *S. aureus* clone, leading to an expansion of its spectrum of action and an increase in its ability to cause severe infections [3–8, 11, 12]. In this study, we investigated, for the first time with whole genome sequencing technology, changes in the prophage content of a cohort of ST398 BSI isolates recovered during a continuous survey conducted between 2000 and 2015 in the same health centers [10]. An analysis of the MGEs of 22 representative isolates, including 35 prophages, provided insights into the ongoing evolution of this human-adapted subpopulation of ST398 isolates.

As expected, the core genomes of representative BSI isolates from the 9-year-period cohort were highly similar, confirming that this ST398 subpopulation probably emerged recently [11]. However, with the exception of φ3-prophages, which are generally considered as a signature of human niche adaptation [37] and carried by most BSI isolates, the accessory gene pool differed considerably between these isolates. In particular, they harbored a number of MGEs or MGE remnants usually found in animal-associated isolates (i.e., *SCCmec* XI element or *Tn916* transposon) [38], enabling the probable origin of these isolates to be genetically traced to an animal setting. From these data, we can deduce that the increasing incidence of ST398-associated BSI is not due to a single epidemic event. Instead, it likely resulted when a number of isolates initially present in animal niches acquired the φ3-prophage that contributed to their adaptation to humans as a new host. The fact that we identified remnant elements specific to the animal environment also suggests that the loss of these animal-associated factors does not impair ST398’s ability to colonize or infect humans. Some of these elements could therefore be considered as animal-specific in an environment shared by animals and humans.

The genome of one BSI isolate appeared highly similar to that of an isolate obtained from an infected animal, due to the presence of four animal-associated MGEs and the absence of the φ3-prophage. This isolate was recovered from a 2-month-old baby diagnosed with a BSI of pulmonary origin in 2014. All previous human infections associated with animal-associated isolates involved farmers and veterinary surgeons in close contact with asymptomatic colonized pigs [1, 2]. However, in this case, the infection was caused by an “animal-like” isolate in the absence of any link to an animal environment for the baby or other family members. The reasons for the infection of this young patient with this isolate remain unclear and further studies are required to investigate new routes of transmission for human infections caused by isolates responsible for infections in animals. 

A detailed analysis of the complete sequences of 35 prophages revealed the presence of a number of genes potentially involved in *S. aureus* pathogenesis, including genes encoding putative virulence factors, factors involved in cell division, biofilm formation, bacterial persistence, and the rapid adaptation of bacteria to challenging environments, or factors implicated in bacterial resistance to foreign DNA uptake. Most of these factors have been shown to be associated with greater virulence or immune evasion in animal models of staphylococcal infections [26–36]. We hypothesize that lysogeny has played a significant role in increasing the ability of the ST398 clone to cause infections in humans and to become rapidly established in hospital environments. We are currently testing this hypothesis using an in vivo model to further investigate the functional impact of lysogeny on the successful adaptation of this lineage to humans.

Isolates from the ST398 lineage lack the type I restriction system (*hsdS-hsdR*), the main function of which is the limitation of horizontal gene transfer [39, 40]. These isolates therefore should have a particularly strong ability to acquire genetic material following horizontal transfers. By studying the prophage content of the whole cohort of BSI isolates, we found that the prevalence of polylysogeny had increased over the 9-year period. At the time of the first BSI cases and during the subsequent 5 years, BSI was significantly associated with isolates carrying a single prophage. In contrast, after 2012 most of the diagnosed BSI cases were associated with polylysogenic isolates. In this study, the pool of prophage genes available to BSI isolates increased over time. Depending on the number of prophages carried, lysogenic isolates have access to pools of prophage genes of various degrees of completeness. From our findings, we hypothesize that if the ST398 human-adapted subpopulation is continuing to evolve, it could become more virulent and better adapted to the human clinical settings. In our lab we are testing this hypothesis by conducting further investigations into the functional impact of the prophage genes on the fitness, adaptation and pathogenicity of the bacterial host. To date, reports of human infections with ST398 isolates carrying virulence features or antibiotic resistance determinants remain scarce [38]. However, the increasing number of patients infected or colonized with ST398 isolates in hospital settings, in which dangerous staphylococcal clones are already established, may favor the acquisition of additional genes by this lineage, resulting in a much higher epidemic or virulence potential. In particular the acquisition of multiple resistance determinants would be disastrous and is thus highly feared. Our analysis revealed the presence of putative defense systems limiting horizontal transfer in most of the prophages identified, suggesting that such genetic transfer events are not particularly likely, but nevertheless, this lineage should be closely monitored.

## Conclusion

This study provides the first characterization of the prophages carried by ST398 isolates during their adaptation to humans, after almost a decade of observation. Our molecular data provide strong evidence for the rapid, ongoing evolution of ST398 isolates through various recent, probably independent events. The finding of significant numbers of animal-associated features in the genome of BSI isolates from patients living in animal-free environments suggests that isolates colonizing or infecting animals may have played a major role in the epidemiological changes to this lineage currently observed in the human setting. Based on the significant increase in the prevalence of ST398-BSI and the potential risk of further evolution toward virulence, these results support the continuation of this large-scale survey program.

## Additional files

Additional file 1: Table S2. Prophage DNA fragments detected using Kahankova method (24). (DOCX 736 kb)
Additional file 2: Figure S1. Pairwise comparison of the number of SNPs and mean nucleotide identity of the genomes from 16 BSI isolates and the animal-associated isolate (S0385) recovered from a case of human endocarditis contracted in a livestock environment. (DOCX 158 kb)
Additional file 3: Figure S2. Comparative genomics for the identified prophages from three groups: φ3, MR11-like and others. The graph on the left illustrates the comparative genomics of the prophages. From outside to inside: ring 1, COG classification (see COG definitions in supplementary table S1) of ORFs in ring 2; rings 2 and 3, ORFs; ring 4, COG classification of ORFs in ring 3; rings 5 to 19 (in blue), the φ3 prophage genomes; rings 20 to 24 (in green), the genomes of prophages from “others” group; rings 25 to 30 (in red), the MR11-like prophage genomes; rings 31 to 33, the %GC content of the reference genome. The graph on the right shows the comparative genomics of the core prophage genomes (percent identity of the core genome of the prophages in each group). (DOCX 3531 kb)
Additional file 4: Table S1. COG legend definitions. (DOCX 22 kb)

## Abbreviations

BSI: Bloodstream Infection
CV: Composition Vector
MGE: Multiple mobile genetic element
MLST: Multi-locus sequence typing
SCC*mec*: Staphylococcal cassette chromosome mec
SNP: Single Nucleotide polymorphism
ST: Sequence Type
WGS: Whole-genome sequencing
