# Supplementary figures and images for: Prophages and adaptation of Staphylococcus aureus ST398 to the human clinic
Source: BMC Genomics. 2017 Feb 6;18:133. doi: 10.1186/s12864-017-3516-x (PMC5294865; doi:10.1186/s12864-017-3516-x)

**Additional file 1: Table S2**. Prophage DNA fragments detected using Kahankova method (24)


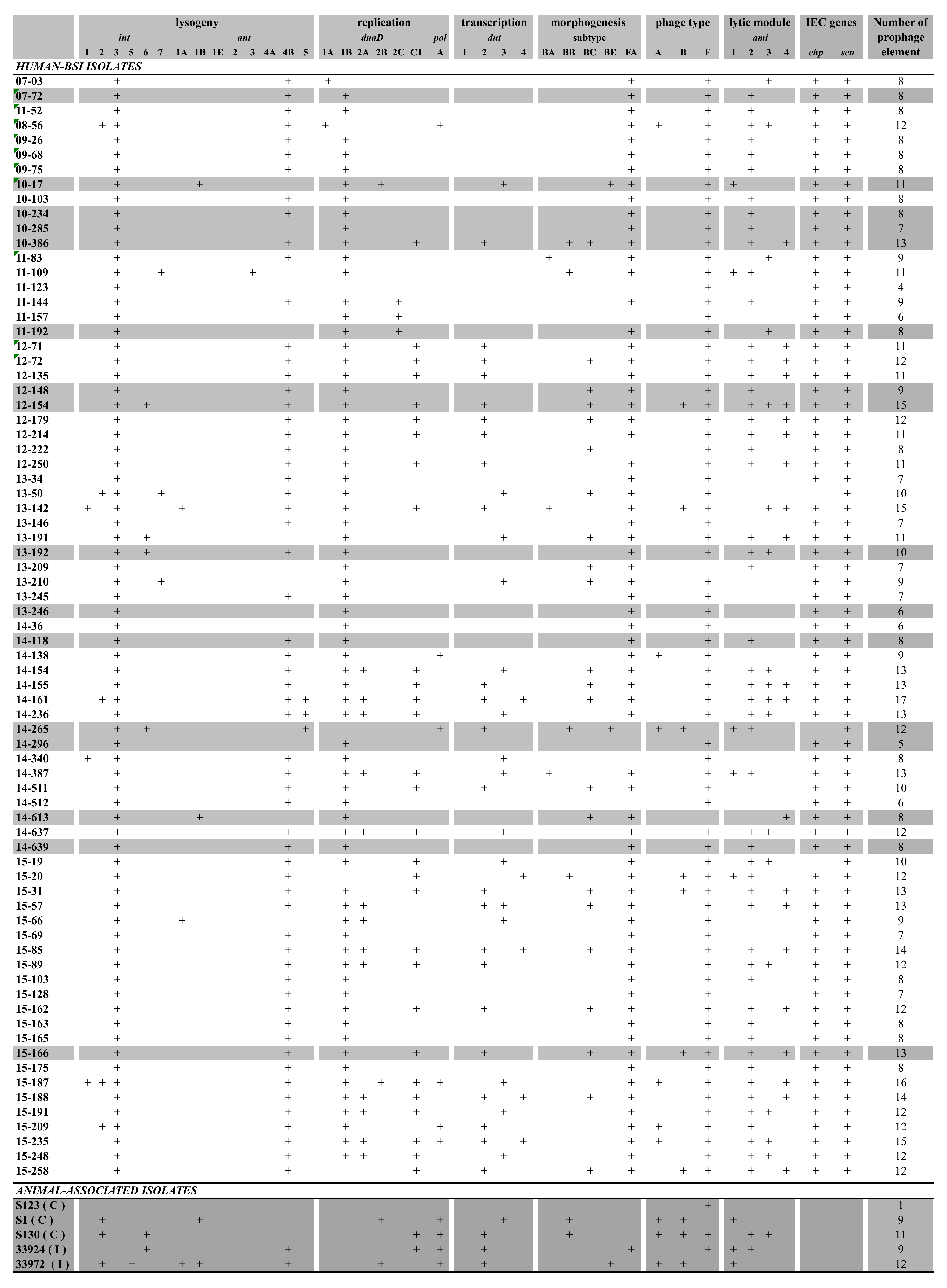

Supplement: Additional file 1: Table S2. — Prophage DNA fragments detected using Kahankova method (24). (DOCX 736 kb) [file 12864_2017_3516_MOESM1_ESM.docx]
