# Supplementary material for: Prophages and adaptation of Staphylococcus aureus ST398 to the human clinic
Source: BMC Genomics. 2017 Feb 6;18:133. doi: 10.1186/s12864-017-3516-x (PMC5294865; doi:10.1186/s12864-017-3516-x)
Supplement: Additional file 3: Figure S2. — Comparative genomics for the identified prophages from three groups: φ3, MR11-like and others. The graph on the left illustrates the comparative genomics of the prophages. From outside to inside: ring 1, COG classification (see COG definitions in supplementary table S1) of ORFs in ring 2; rings 2 and 3, ORFs; ring 4, COG classification of ORFs in ring 3; rings 5 to 19 (in blue), the φ3 prophage genomes; rings 20 to 24 (in green), the genomes of prophages from “others” group; rings 25 to 30 (in red), the MR11-like prophage genomes; rings 31 to 33, the %GC content of the reference genome. The graph on the right shows the comparative genomics of the core prophage genomes (percent identity of the core genome of the prophages in each group). (DOCX 3531 kb) [file 12864_2017_3516_MOESM3_ESM.docx]

**Prophages of the φ3 group**

| **Comparative genomics of the φ3 prophages (in blue), MR11-like prophages (in red), and other prophages (in green).** | **Percent identity of the core genome of φ3 prophages**  **This group has a core genome of 25 genes** |
| --- | --- |
| 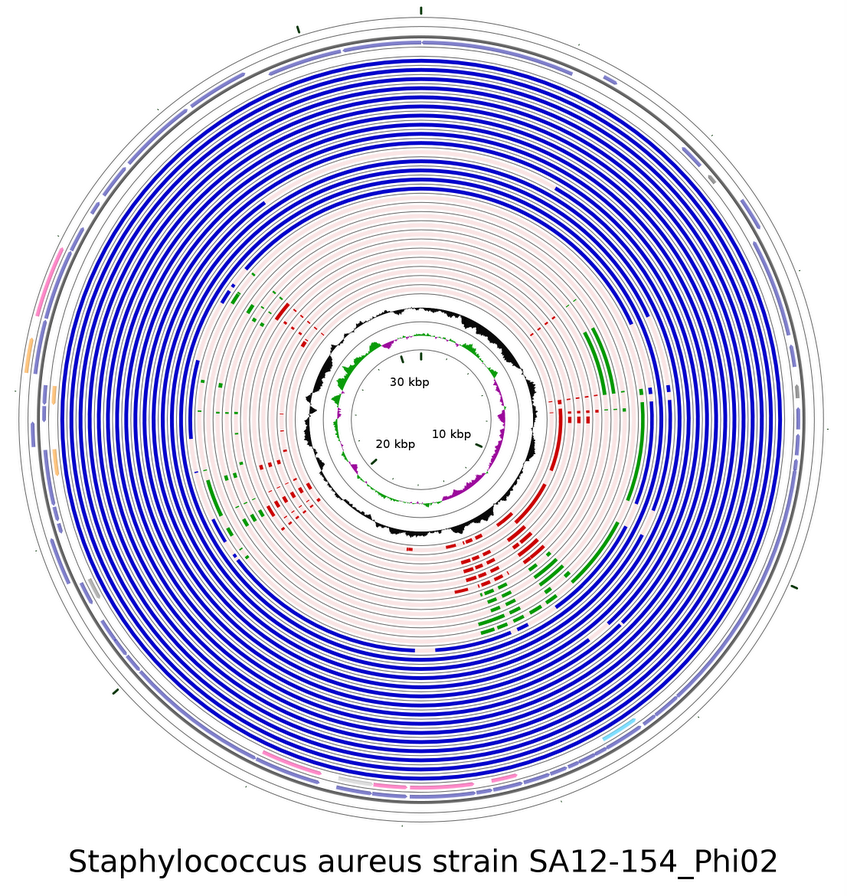  **The genome of the 12-154 phi02 prophage is used as reference.** | **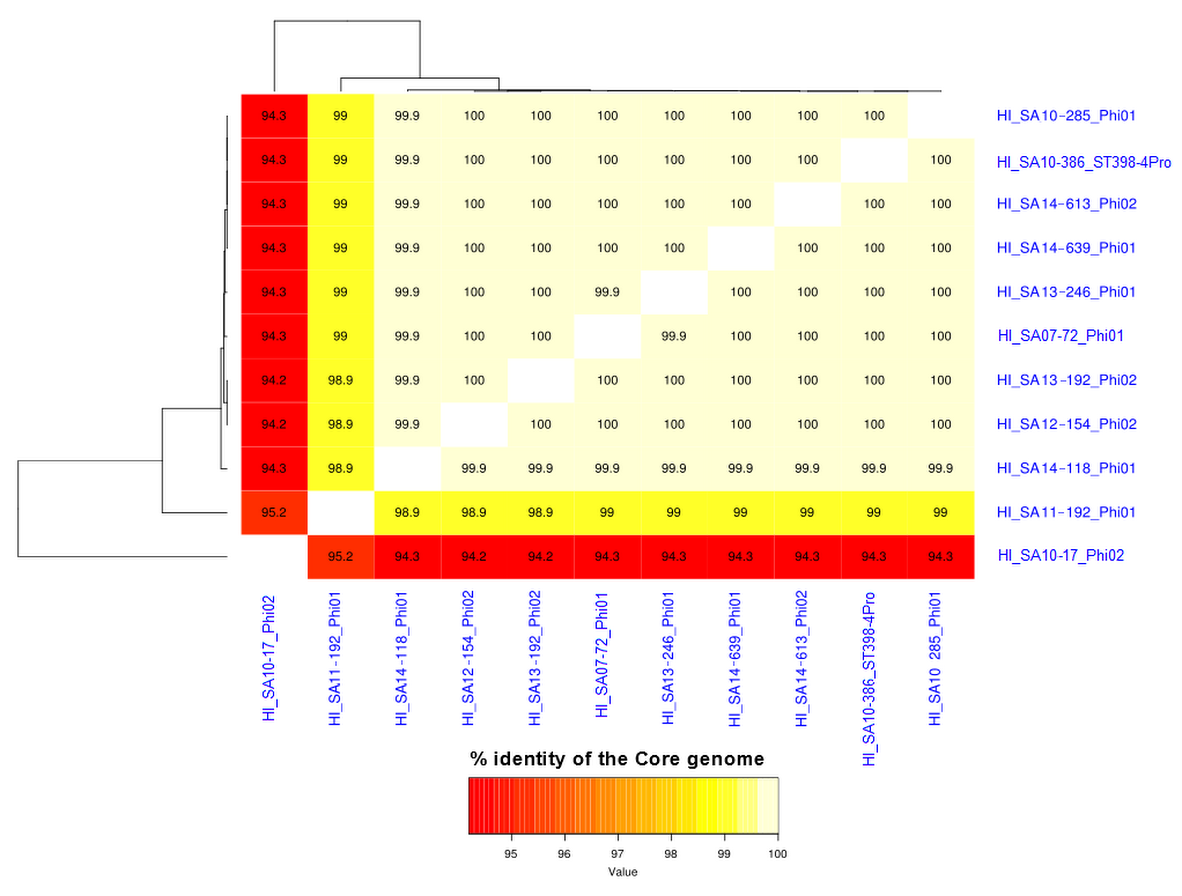** |

**Prophages of MR11-like group**

| **Comparative genomics of the φ3 prophages (in blue), MR11-like prophages (in red), and other prophages (in green).** | **Percent identity of the core genome of prophages of MR11-like group. This group has a core genome of 15 genes** |
| --- | --- |
| 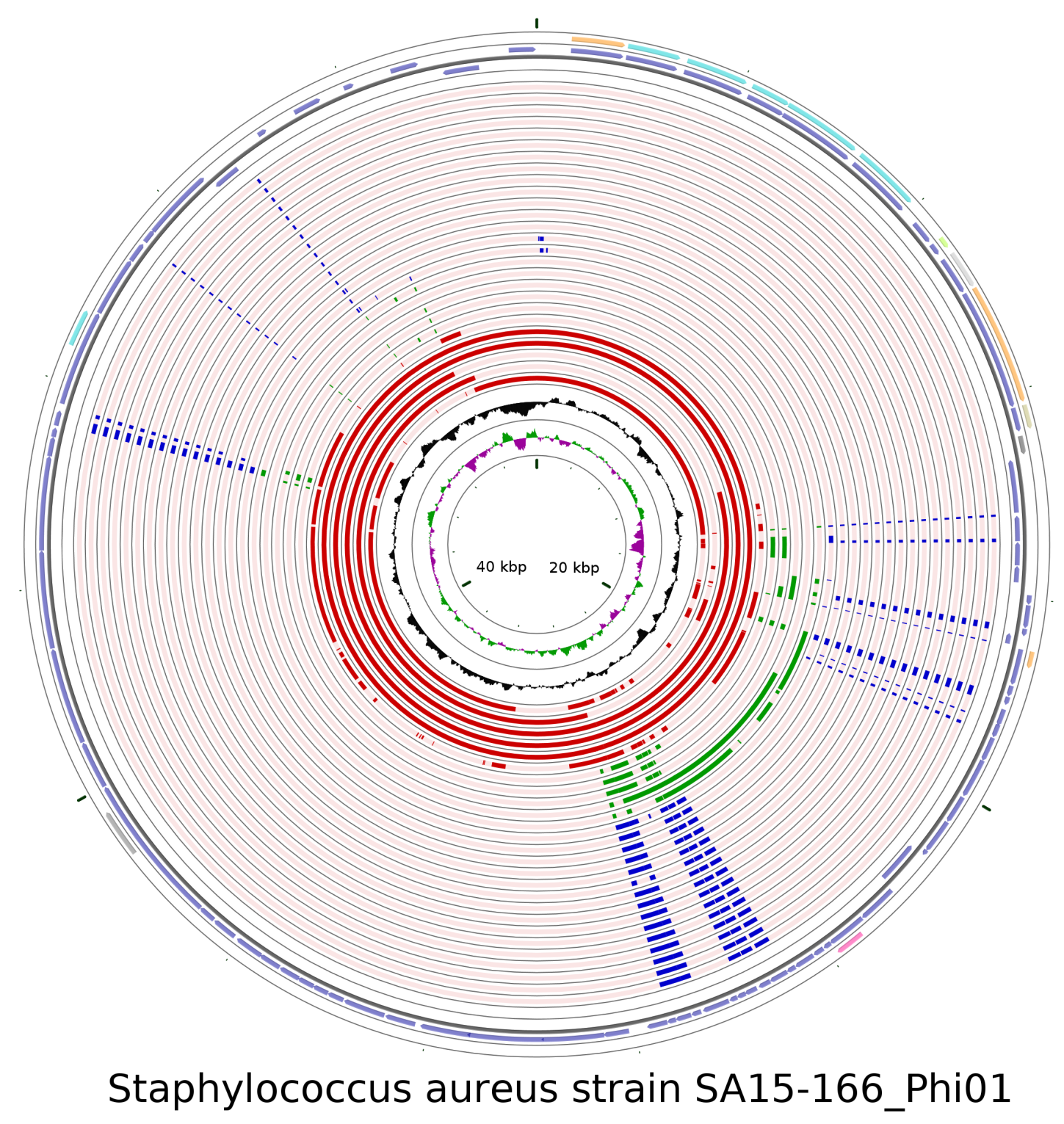  **The genome of the 15-166 phi01 prophage is used as a reference.** | **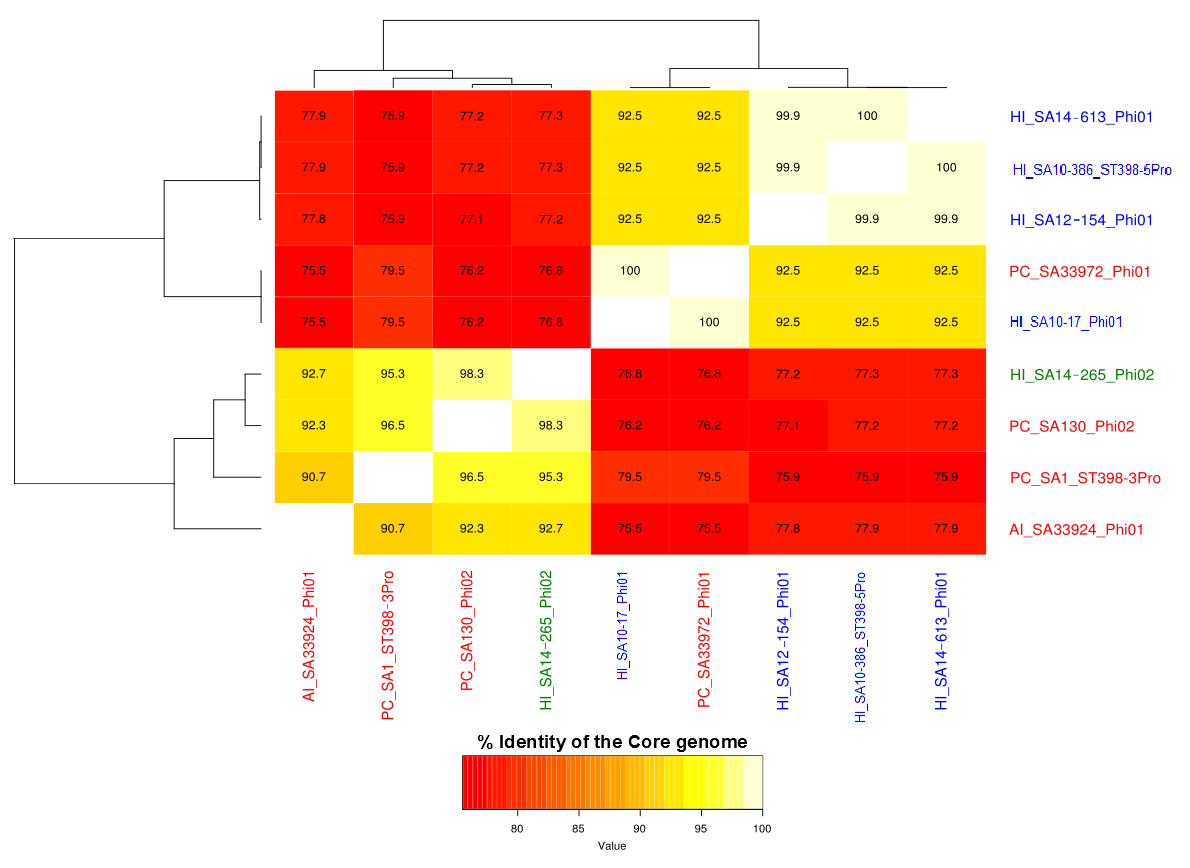** |

**Prophages of the “others” group.**

| **Comparative genomics of the φ3 prophages (in blue), MR11-like prophages (in red), and other prophages (in green).** | **Percent identity of the core genome of prophages of the “others” group. This group has a core genome of 3 genes** |
| --- | --- |
| 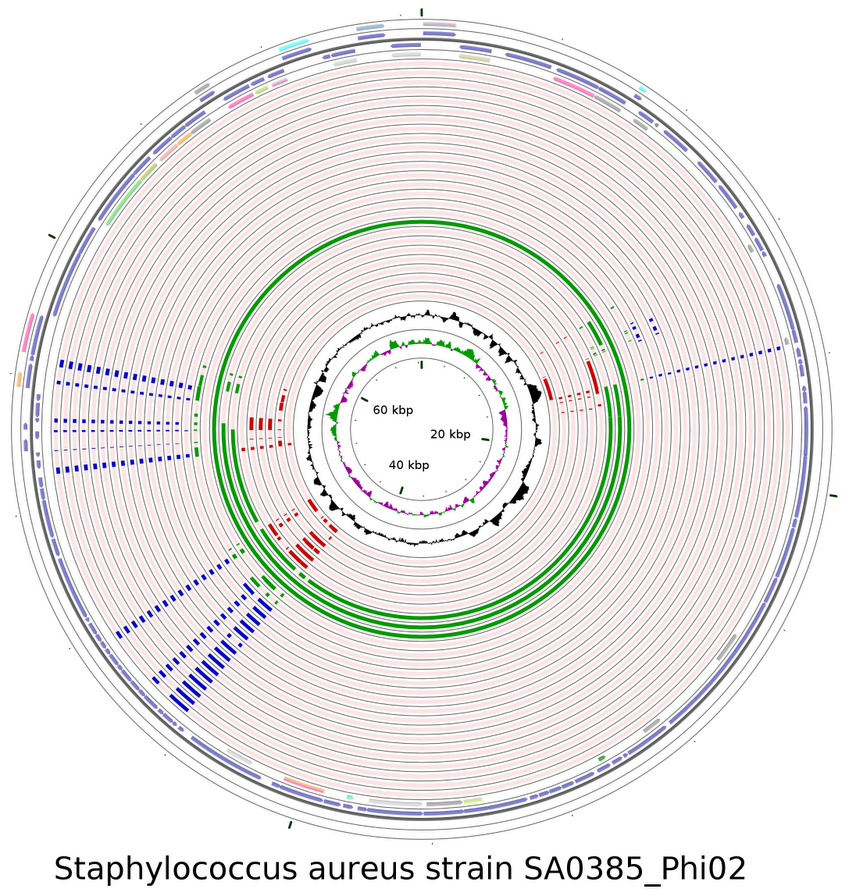  **The genome of the s0385 phi02 prophage is used as reference.** | **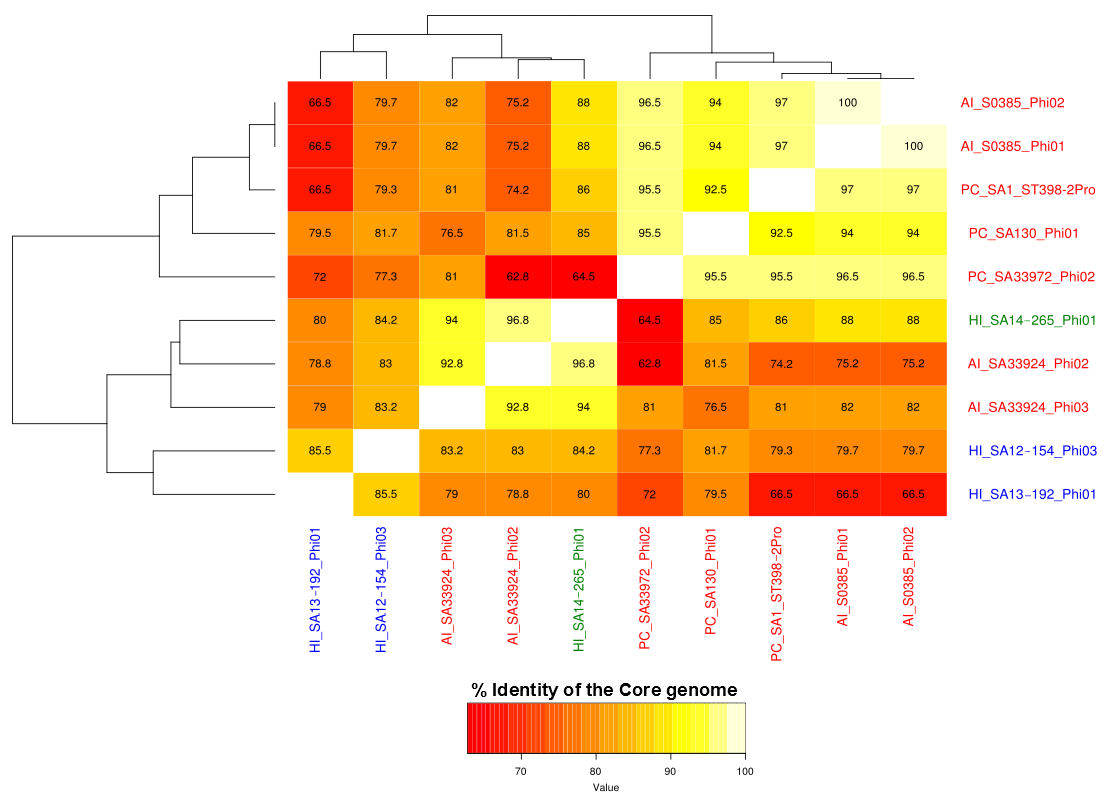** |
