# Supplementary material for: Prophages and adaptation of Staphylococcus aureus ST398 to the human clinic
Source: BMC Genomics. 2017 Feb 6;18:133. doi: 10.1186/s12864-017-3516-x (PMC5294865; doi:10.1186/s12864-017-3516-x)
Supplement: Additional file 4: Table S1. — COG legend definitions. (DOCX 22 kb) [file 12864_2017_3516_MOESM4_ESM.docx]

**Additional file 4: Table S1**. COG legend definitions

| **Abbreviation** | **Color code** | **Definitions** |
| --- | --- | --- |
| **A** | Red | RNA processing and modification |
| **B** | Tomato | Chromatin structure and dynamics |
| **J** | Light coral | Translation, ribosomal structure and biogenesis |
| **K** | Dark orange | Transcription |
| **L** | Deep pink | Replication, recombination and repair |
| **D** | Khaki | Cell cycle control, cell division, chromosome partitioning |
| **O** | Dark khaki | Post-translational modification, protein turnover, and chaperones |
| **M** | Olive drab | Cell wall/membrane/envelope biogenesis |
| **N** | Forest green | Cell motility |
| **P** | Yellow green | Inorganic ion transport and metabolism |
| **T** | Lime green | Signal transduction mechanisms |
| **U** | Green yellow | Intracellular trafficking, secretion, and vesicular transport |
| **V** | Medium spring green | Defense mechanisms |
| **W** | Dark sea green | Extracellular structures (this doesn't appear in reference database) |
| **Y** | Medium sea green | Nuclear structure (this appears once in reference database) |
| **Z** | Yellow | Cytoskeleton |
| **C** | Cyan | Energy production and conversion |
| **G** | Dark turquoise | Carbohydrate transport and metabolism |
| **E** | Steel blue | Amino acid transport and metabolism |
| **F** | Deep sky blue | Nucleotide transport and metabolism |
| **H** | Blue | Coenzyme transport and metabolism |
| **I** | Slate blue | Lipid transport and metabolism |
| **Q** | Navy | Secondary metabolite biosynthesis, transport, and catabolism |
| **R** | Gray | General function prediction only (examples include "Predicted thioesterase", "Predicted ATPase") |
| **S** | Dark gray | Function unknown (examples include "Uncharacterized conserved protein", "Predicted small secreted protein") |
| **Unknown** | White | Not assigned a COG letter because protein is not similar to any COG |
