# Supplementary material for: Prophages and adaptation of Staphylococcus aureus ST398 to the human clinic
Source: BMC Genomics. 2017 Feb 6;18:133. doi: 10.1186/s12864-017-3516-x (PMC5294865; doi:10.1186/s12864-017-3516-x)
Supplement: Supplementary file 4 [file 12864_2017_3516_MOESM2_ESM.docx]

Close genomes — Remote genomes

	SA10-17	SA0385	SA14-265	SA11-192	SA13-192	SA14-118	SA07-72	SA10-234	SA10-285	SA10-386	SA12-148	SA12-154	SA13-246	SA14-296	SA14-613	SA14-639	SA15-166
**SA10-17**		99.82	99.78	99.81	99.77	99.8	99.82	99.79	99.8	99.73	99.81	99.74	99.8	99.8	99.75	99.79	99.72
**SA0385**	628		99.84	99.89	99.85	99.88	99.91	99.89	99.91	99.88	99.9	99.9	99.91	99.9	99.9	99.88	99.87
**SA14-265**	692	451		99.91	99.86	99.89	99.92	99.91	99.91	99.85	99.9	99.84	99.9	99.91	99.84	99.9	99.83
**SA11-192**	791	520	414		99.85	99.89	99.92	99.91	99.91	99.9	99.9	99.9	99.91	99.9	99.89	99.89	99.88
**SA13-192**	796	549	459	338		99.89	99.89	99.88	99.88	99.86	99.88	99.84	99.87	99.88	99.87	99.87	99.85
**SA14-118**	815	549	439	351	139		99.93	99.92	99.91	99.92	99.91	99.91	99.92	99.93	99.91	99.91	99.91
**SA07-72**	781	450	413	278	206	228		99.94	99.94	99.94	99.93	99.94	99.94	99.94	99.94	99.94	99.93
**SA10-234**	860	516	451	306	252	263	226		99.94	99.94	99.94	99.93	99.93	99.94	99.93	99.94	99.93
**SA10-285**	769	435	397	246	215	231	199	225		99.93	99.95	99.92	99.93	99.93	99.93	99.92	99.9
**SA10-386**	988	540	525	316	284	272	239	173	224		99.92	99.94	99.93	99.93	99.94	99.93	99.93
**SA12-148**	783	446	414	261	235	244	213	238	35	242		99.91	99.92	99.92	99.93	99.92	99.91
**SA12-154**	931	487	520	286	303	254	215	154	213	153	226		99.93	99.92	99.93	99.93	99.91
**SA13-246**	782	445	414	282	236	244	209	231	202	233	215	219		99.93	99.93	99.93	99.91
**SA14-296**	811	469	430	303	250	262	226	231	218	248	232	221	224		99.93	99.94	99.92
**SA14-613**	971	516	538	325	290	283	247	182	241	178	254	165	248	247		99.93	99.91
**SA14-639**	850	543	441	385	339	338	219	157	221	160	234	138	225	232	166		99.93
**SA15-166**	1008	555	543	334	303	282	250	184	247	180	261	172	254	252	195	171	

ANI

Number of SNPs

ANI: Average nucleotide identity of the genome

SNP: Single nucleotide polymorphisms
